# AIM2 inhibits autophagy and IFN-β production during *M. bovis* infection

**DOI:** 10.18632/oncotarget.10503

**Published:** 2016-07-09

**Authors:** Chunfa Liu, Ruichao Yue, Yang Yang, Yongyong Cui, Lifeng Yang, Deming Zhao, Xiangmei Zhou

**Affiliations:** ^1^ State Key Laboratories for Agrobiotechnology, Key Laboratory of Animal Epidemiology and Zoonosis, Ministry of Agriculture, National Animal Transmissible Spongiform Encephalopathy Laboratory, College of Veterinary Medicine, China Agricultural University, Beijing, China; ^2^ College of Animal Sciences and Technology, Zhejiang A&F University, Lin'an, China

**Keywords:** M. bovis, autophagy, AIM2 inflammasome, STING, Immunology and Microbiology Section, Immune response, Immunity

## Abstract

Mycobacteria can trigger the AIM2 inflammasome, autophagy activation and type-I interferon release, which are both activated by cytosolic DNA. We have recently demonstrated that activation of the AIM2 inflammasome during *M. bovis* infection is the result of mycobacterial translocation into the cytosol. To elucidate the effects of inflammasome activation on autophagy, we investigated the role of the AIM2 inflammasome from macrophages infected with a virulent strain of *M. bovis*. The results showed that the *M. bovis*-induced AIM2 inflammasome activation decreases autophagy in immortalized and primary murine macrophages. This relied on the inflammasome sensor AIM2 which conjugates with cytosolic DNA to inhibit the STING-dependent pathway involved in selective autophagy and interferon-β release in *Mycobacterium*-infected macrophages. These results suggest that the AIM2 cytosolic DNA sensor may conjugate competitively with cytosolic *M. bovis* DNA to restrict *M. bovis* induced STING-TBK1-dependent autophagy activation and IFN-β secretion.

## INTRODUCTION

*Mycobacterium bovis* (*M. bovis*) is a member of the *Mycobacterium tuberculosis* (*Mtb*) complex and is responsible for bovine tuberculosis and an associated major zoonotic threat to human health. *M. bovis* and *Mtb* are genetically >99% identical and induce similar disease profiles and host responses upon infection [1]. *Mtb* is an intra-cellular pathogen that resides predominantly within macrophages and which utilizes a number of mechanisms to avoid being eliminated and to facilitate survival and replication [2–4].

Autophagy (macroautophagy) is a highly conserved homeostatic process whereby cytosolic macro-molecules, cytoplasmic organelles, some pathogens and immunological mediators are sequestered by a double membrane structure called the autophagosome for subsequent transfer to lysosomes and degradation [5]. Induction of autophagy mediates mycobacterial clearance in infected macrophages by increasing acidification and maturation of mycobacterial phagosomes [6]. Many factors have been shown to inhibit mycobacterial survival by inducing autophagy [7–11]. Mice with an Atg5 (autophagy-related protein 5) deletion in the myeloid lineage are more susceptible to *Mtb* infection [12]. These results illustrate the important role of autophagy in controlling mycobacterial damage to the host. Inducing autophagy by exogenous agents has a negative effect on pathogen survival. However, we know less about the induction mechanism of autophagy in mycobacterial infection although some studies have demonstrated *Mtb* can activate autophagy by recognition of extracellular bacterial DNA in the STING-dependent (stimulator of interferon genes) cytosolic pathway [13].

AIM2 (absent in melanoma 2), a cytosolic sensor for double-stranded DNA (dsDNA), activates the inflammasome with ASC (apoptosis-associated speck-like protein containing a caspase recruitment domain) that leads to caspase-1 cleavage [14, 15]. Several cytosolic bacterial pathogens have been demonstrated to be involved in AIM2 inflammasome activation [16–19]. *Mtb* and *M. leprae* can translocate from phagolysomes to the cytosol of myeloid cells in a RD1 (region of difference-1)-dependent manner [20]. These results are consistent with our results of *M. bovis*-induced activation of the AIM2 inflammasome following translocation of the bacteria from the phagosome to the cytosol [16]. In contrast, several reports have demonstrated that activation of autophagy by double-stranded DNA (dsDNA) from the avirulent BCG does not possess RD1 in an AIM2-dependent manner [21, 22].

STING (stimulator of interferon genes), also known as MITA/MPYS/ERIS/TMEM173, is an important signal adapter molecule in the innate immune system. STING/TBK1/IRF3 signaling pathways play an important role in the process of expression of type I interferon and autophagy induction in phagocytes infected by *Mtb* [13, 23, 24]. Recent studies have shown that cGAS (cyclic GMP-AMP synthase), a dsDNA sensor, participates in the innate immune response induced by bacterial dsDNA release mediated by the ESX-1 secretion system *via* cGAS-STING pathway [25].

In this study, we investigated the effect of the AIM2 inflammasome on autophagy in murine macrophages upon *M. bovis* infection. Our data indicate that the AIM2 inflammasome sensor inhibits autophagy and IFN-β production during *M.bovis* infection of macrophages.

## RESULTS

### *M. bovis* induces autophagy in murine macrophages

**Figure 1:**
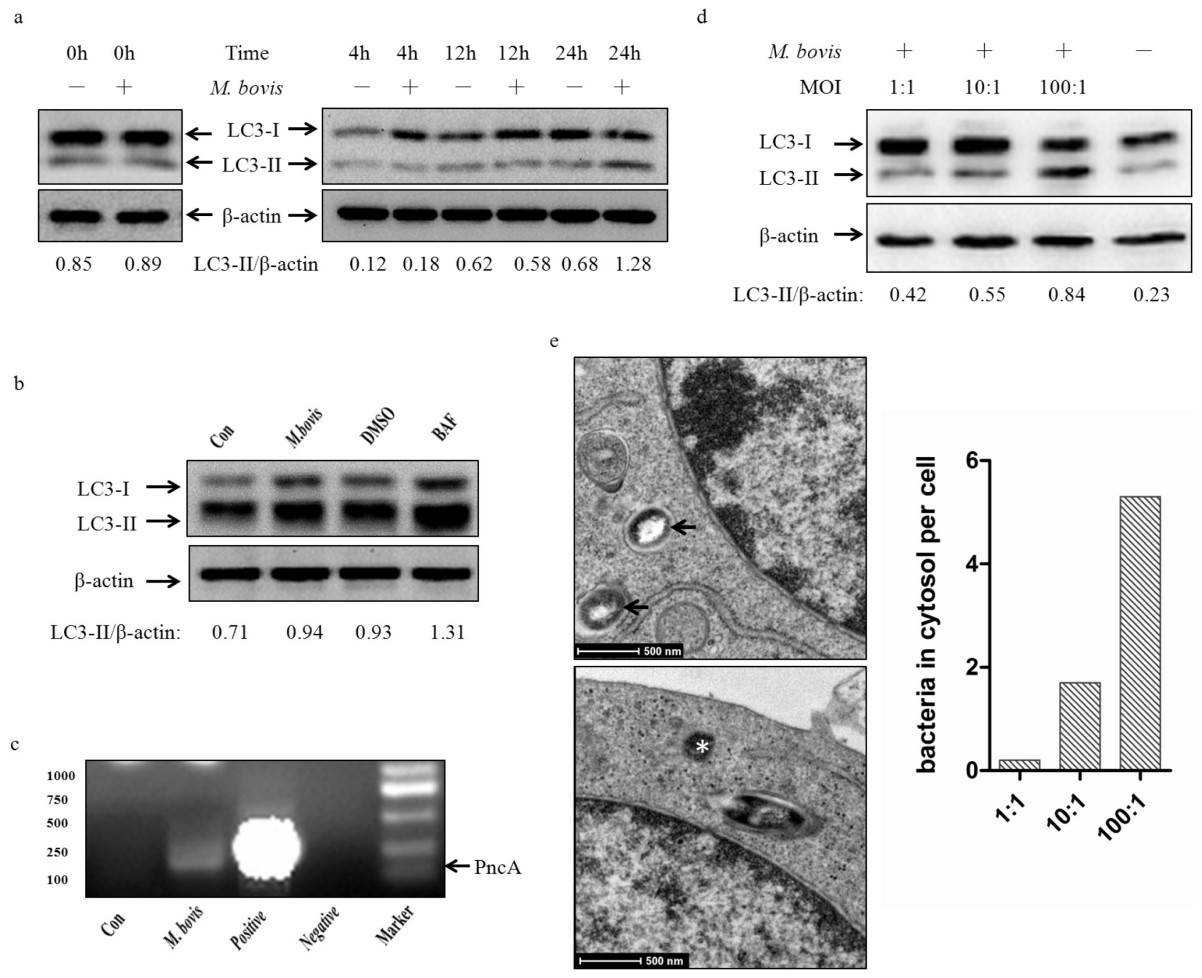
M. *bovis* induces autophagy in murine macrophages **a.** Protein level of LC3-II were analyzed using western blotting in murine BMDMs infected with *M. bovis* (MOI 10) for various times (0h, 4h, 12h, 24h) with the LC3-II/β-actin ratio shown below. **b.** Protein level of LC3-II were analyzed using western blotting in murine BMDMs treated with *M. bovis* (MOI 10) (2^nd^ lane), *M. bovis* (MOI 10) and DMSO (DMSO) (3^rd^ lane), or *M. bovis* (MOI 10) and Bafilomycin A1 (BAF) (4^th^ lane) at 24h post-infection with the LC3-II/β-actin ratio shown below. **c.** BMDMs were infected with *M. bovis* (MOI 10) for 3 hours, and bacterial DNA was isolated from purified cytosolic fraction. The target gene *pncA* was amplified by PCR using the specific primers (Forward primer: CTCAGCTGGTCATGTTCCCCAT, Reverse primer: CGGTGTGCCGGAGAAGCCG). A 294bp fragment was specifically amplified from *M. bovis*. ‘Con’ is water, ‘Negative’ is the DNA isolated from the cells with no infection. **d.** Protein level of LC3-II were analyzed using western blotting in murine BMDMs infected with *M. bovis* at different MOI (1,10,100) after 24h hours with the LC3-II/β-actin ratio shown below. Data were performed three times and expressed as the mean ± SD, and are representative of three separate experiments. **e.** TEM analysis the distribution of bacteria in J774A.1 macrophages infected with *M. bovis* at different MOI (1, 10,100) after 24h hours. Intraphagosomal bacteria are indicted by an arrow and cytosolic bacteria by an asterisk (left). Quantification of bacteria (*n* = 145) located in intraphagosome and cytoplasm of infected mouse macrophages by TEM (right). Abbreviations: MOI, multiplicity of infection; TEM, transmission electron microscopy.

Murine bone marrow monocyte-derived macrophages (BMDMs) were infected with *M. bovis* at a multiplicity of infection (MOI) of 10, and the level of the autophagy marker, microtubules associated protein light chain 3 (LC3) in cell lysate detected by immunoblotting at 0h, 4 h, 12 h, and 24 h post-infection. We found an increase in the LC3-II/β-actin ratio at 24 h (Figure 1a). We used bafilomycin A1 to confirm that the higher expression of LC3-II was not the result of autophagy flux inhibition by *M. bovis* (Figure 1b). Recent studies have shown that cytosolic DNA can activate autophagy [13]. Our previous results suggested that *M. bovis* can escape from the phagosome to the cytosol [16] and we detected *M. bovis* DNA in the cytosol at 24 h post-infection (Figure 1c).

To determine the extent of interaction between the level of LC3-II and cytosolic *M. bovis*, we investigated the effect of bacterial load on autophagy by exposing BMDMs to *M. bovis* at three different MOI (1, 10 and 100) (Figure 1d). Then the numbers of *M. bovis* in the cytosol were examined at 24 h post-infection by TEM at the same three MOI (Figure 1e), and the results showed a positive correlation between LC3-II and cytosolic *M. bovis*.

### The AIM2 inflammasome down-regulates *M. bovis*-induced autophagy

**Figure 2:**
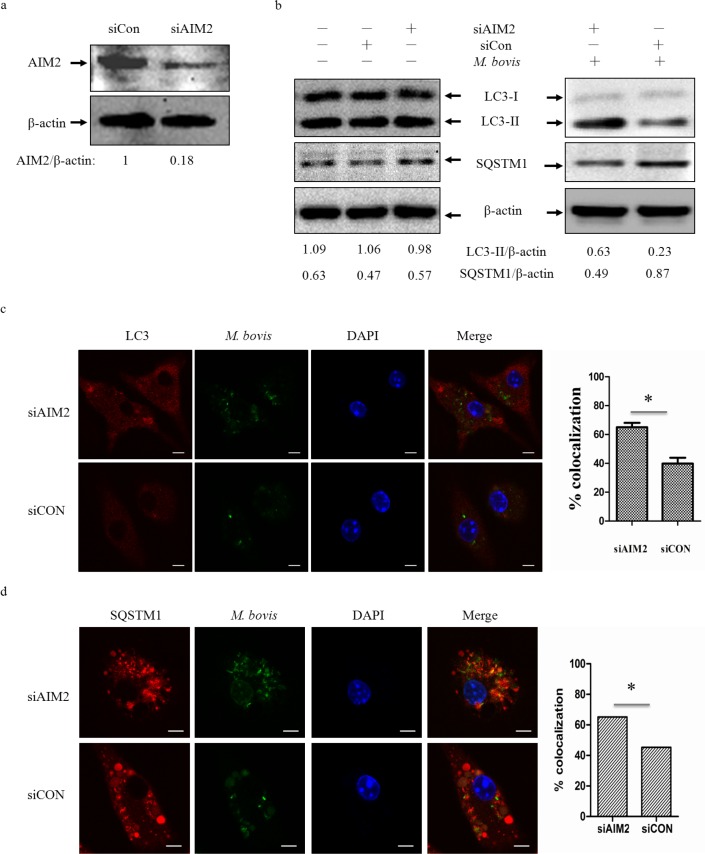
The AIM2 inflammasome down-regulates *M.bovis*-induced autophagy **a.** The knockdown efficiency of AIM2 by small interference RNA (siRNA). Protein level of AIM2 was analyzed using western blotting in J774A.1 macrophages transfected with non-targeting siRNA (siCon), or AIM2-targeting siRNA (siAIM2). The AIM2/β-actin ratios are shown below. **b.** Protein level of autophagic markers LC3-II and SQSTM1 (P62) were analyzed using western blotting in J774A.1 macrophages transfected with siCon or siAIM2 and then infected 4h with *M. bovis* (MOI 10) (right) or no infection (left). The LC3-II/β-actin and SQSTM1/β-actin ratios are shown below. Data were performed three times and expressed as the mean ± SD, and are representative of three separate experiments. *P < 0.05, siAIM2 group *versus* siCON group. **c.**, **d.** Immunofluorescence staining of LC3 (red) (c) or SQSTM1 (red) (d) in murine BMDMs transfected with siCon or siAIM2 were infected with Alexa 488-labeled *M. bovis* (green) (MOI 10) for 24h and the proportion of LC3-positive mycobacteria is shown (right) and the proportion of SQSTM1-positive mycobacteria is shown (right). Nuclei were counterstained with DAPI (blue). Scale bar, 5um. Approximately 120 cells wre used to calculate the percentage of colocalization. The image J software was used for the analysis.

**Figure 3:**
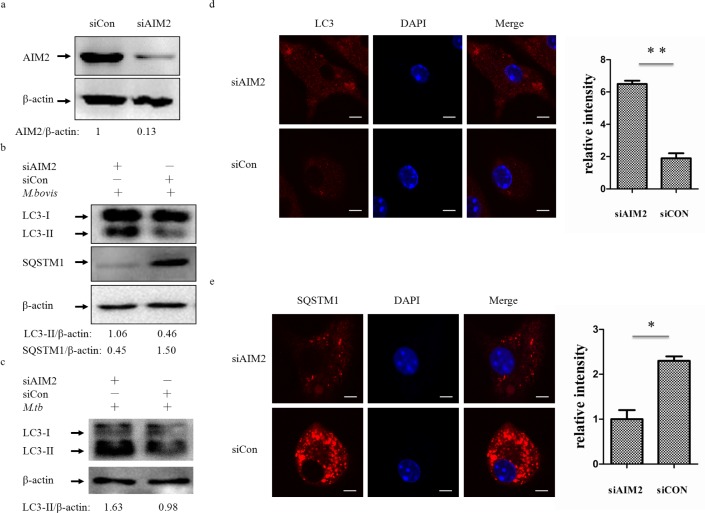
The AIM2 inflammasome down-regulates *M. bovis*-induced autophagy **a.** The knockdown efficiency of AIM2 by small interference RNA (siRNA). Protein level of AIM2 was analyzed using western blotting in BMDMs transfected with siCon or siAIM2. The AIM2/β-actin ratios are shown below. **b.** Protein level of LC3-II and SQSTM1 (P62) were analyzed using western blotting in BMDMs transfected with siCon or siAIM2 and then infected 4h with *M. bovis* (MOI 10). The LC3-II/β-actin and SQSTM1/β-actin ratios are shown below. **c.** Protein level of LC3-II was analyzed using western blotting in murine BMDMs transfected with siCon or siAIM2 and then infected 24h with *Mtb* (MOI 10). The LC3-II/β-actin ratiois are shown below. **d.**, **e.** Immunofluorescence staining of LC3 (red) (d) or SQSTM1 (red) (e) in murine BMDMs transfected with siCon or siAIM2 were infected with *M. bovis* (MOI 10) for 24h and the relative fluorescence intensity of LC3 is shown (d, right) and the relative fluorescence intensity of SQSTM1 is shown (e, right). Nuclei were counterstained with DAPI (blue). Scale bar, 5um. Nearly 120 cells were counted for the relative fluorescence intensity. The image J software was used for the analysis.

To test whether the AIM2 inflammasome have an influence on autophagy in *M. bovis*-infected macrophages, we first knocked down the expression of AIM2 in J774A.1 murine macrophages using small interference RNA (siRNA) (Figure 2a) and proved that siRNA has no effect on LC3-II and the autophagic adaptor p62 protein (sequestosome 1 protein; SQSTM1; A170; ZIP) expression (Figure 2b left). Downregulation of AIM2 expression increased LC3-II and reduced the autophagic adaptor p62 protein [26] at 4h post-infection(Figure 2b right). To confirm our result, we tested the role of AIM2 by knocking down the gene using siRNA in BMDMs (Figure 3a) with the same result observed at 24h (Figure 3b). The difference in response with time between J774A.1 and BMDMs may derive from the different origin of the macrophages. Repetition of the BMDM experiment with a wild virulent *Mtb* strain isolated from cattle produced the same result with LC3-II (Figure 3c).

To further elucidate the influence of the AIM2 inflammasome on *M. bovis*-induced autophagy, immunofluorescence was used to detect the amounts of LC3 and SQSTM1/p62 in BMDMs at 24 h post-infection and we saw increased LC3 and decreased SQSTM1/p62 (Figure 3d and 3e). These results showed that the level of autophagy increased in macrophages with AIM2 knockdown. To exam whether the increased autophagy associated with bacteria, we then examined the co-localization of LC3 and SQSTM1/p62 with *M. bovis* in BMDMs. More *M. bovis* was associated with SQSTM1/p62 and LC3 following AIM2 knockdown (Figure 2c and 2d). Taken together, the AIM2 inflammasome appears to have an inhibitory effect on *M. bovis*-induced autophagy.

### The reduction on *M. bovis*-induced autophagy depends on the inflammasome sensor

**Figure 4:**
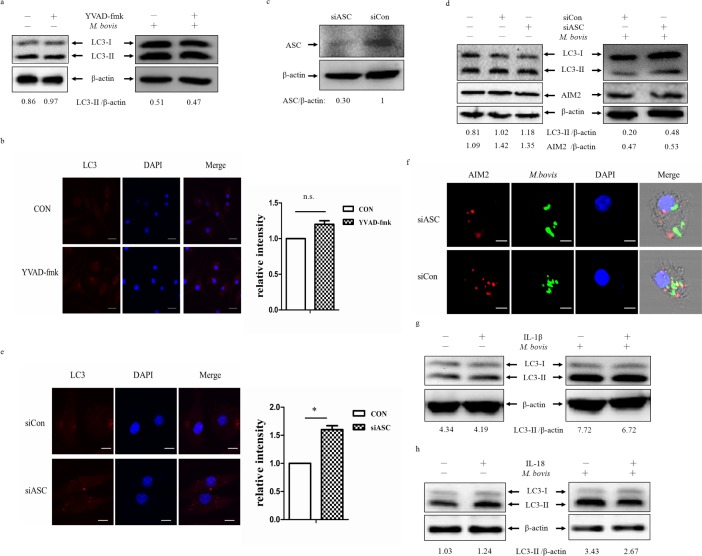
The reduction on *M. bovis*-induced autophagy depends on the inflammasome sensor **a.** Protein level of LC3-II was analyzed using western blotting in BMDMs (left) or BMDMs infected with *M. bovis* (MOI 10) for 24h (right) in presence or absence of the caspase-1 inhibitor VYAD-fmk. The LC3-II/β-actin ratios are shown below. **b.** Immunostain analysis of LC3 (red) of BMDMs infected with *M. bovis* (MOI 10) for 24h in presence or absence of VYAD-fmk and relative fluorescence intensity was shown (right). Nuclei were counterstained with DAPI (blue). Scale bar, 5um. The image J software was used for the analysis. **c.** The knockdown efficiency of ASC by small interference RNA (siRNA). Protein level of ASC was analyzed using western blotting in murine BMDMs transfected with control non-targeting siRNA (siCon), or ASC-targeting siRNA (siASC). The ASC/β-actin ratio is shown below. **d.** Western blot analysis of AIM2 and LC3-II of BMDMs transfected with siCon or siASC and BMDMs transfected with siCon or siASC and then infected with *M. bovis* (MOI 10) for 24h. The AIM2/β-actin ratios and LC3-II/β-actin ratios are shown below. **e.** Immunostain analysis of LC3 (red) of BMDMs transfected with siCon or siASC and then infected with *M. bovis* (MOI 10) for 24h, the relative fluorescence intensity was shown (right). Nuclei were counterstained with DAPI (blue). Scale bar, 5um. Nearly 120 cells wre counted for the relative fluorescence intensity. The image J software was used for the analysis. **f.** Immunofluorescence staining of AIM2 (red) of murine BMDMs treated with ASC siRNA and then infected with Alexa 488-labeled *M. bovis* (green) (MOI 10) for 24h. Nuclei were counterstained with DAPI (blue) Scale bar, 5um. **g.** Western blot analysis of LC3-II of BMDMs (left) or BMDMs infected with *M. bovis* (MOI 10) for 24h (right) in presence or absence of IL-1β (upper band) or IL-18 (below band) neutralizing antibody. The LC3-II/β-actin ratios are shown below. Data were performed three times and expressed as the mean ± SD, and are representative of three separate experiments.

The AIM2 inflammasome is composed of ASC, pro-caspase-1 and AIM2. The gene expression of AIM2 affects the cleavage of caspase-1 during *M. bovis* infection [16]. We tested LC3-II expression in BMDMs after *M. bovis* infection in the presence or absence of the caspase-1 specific inhibitor, Z-YVAD-fmk (Figure 4a and 4b). The data indicated that there were no obvious differences in the level of autophagy.

The AIM2 inflammasome assembly requires PYD-PYD interaction between ASC and AIM2. To test the effect of ASC, we used ASC siRNA to knock down its expression (Figure 4c), and about 70% of the protein expression were inhibited. Frist, we tested the siRNA has no effect on LC3-II and AIM2 (Figure 4d left). Then we examined LC3 expression in BMDMs by siASC knock-down, and down-regulation of ASC lead to increased autophagy after 24 h post-infection (Figure 4d right and 4e). Although we did not obsereve any variation in the expression of AIM2 in BMDMs treated with ASC-targeting siRNA and non-targeting siRNA (Figure 4d), we detected less co-localization of AIM2 and *M. bovis* after transfected with siASC (Figure 4f). The recongnization of *M. bovis* by AIM2 was indirectly impaired by ASC. Then, we detected whether autophagy here depended on IL-1β and IL-18 production. Neutralization of IL-1β or IL-18 had no effect on LC3 expression (Figure 4g). Thus, the inhibition of autophagy by activation of the AIM2 inflammasome following *M. bovis* infection was dependent on the inflammasome sensor but did not require complete assembly of the inflammasome.

### AIM2 inhibits co-localization of STING and *M. bovis*

**Figure 5:**
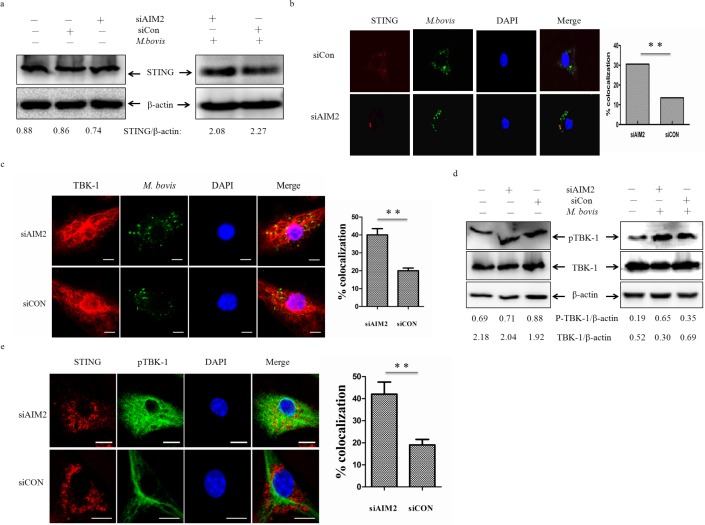
AIM2 inhibits co-localization of STING and *M. bovis* **a.** Protein level of STING was analyzed using western blotting in murine BMDMs transfected with siCon or siAIM2 and then infected 24h with *M. bovis* (MOI 10) (right) or no infection (left). The STING/β-actin ratios are shown below. **b.**, **c.** Immunofluorescence staining of STING (red) (b) and TBK-1 (red) (c) of murine BMDMs infected with Alexa 488-labeled *M. bovis* (green) (MOI 10) for 24h (left), the proportion of STING- and TBK-1-positive mycobacteria is shown (right). Nuclei were counterstained with DAPI (blue). Scale bar, 5um. Approximately 120 cells were used to calculate the colocalization proportion. The image J software was used for the analysis. **d.** Protein level of TBK1 and phosphorylated TBK-1 (p-TBK1) were analyzed using western blotting in murine BMDMs transfected with siCon or siAIM2 and then infected 24h with *M.bovis* (MOI 10) (right) or no infection (left). The TBK1/β-actin and p-TBK1/β-actin ratios are shown below. **e.** Murine BMDMs were infected *M. bovis* (MOI 10) for 24h and immunostained with anti-STING (red) and anti-pTBK-1 (blue) antibody and the percentage of co-localization was shown (right). Nuclei were counterstained with DAPI (blue). Approximately 120 cells are used to calculate the percentage of colocalization. Scale bar, 5um. The image J software was used for the analysis. Data were performed three times and expressed as the mean ± SD, and are representative of three separate experiments. **P < 0.01, siAIM2 group *versus* siCON group.

To our knowledge, recognition of extra-cellular bacterial DNA by the STING-dependent cytosolic pathway plays an important role in *Mtb* targeting by autophagosomes [13]. The innate immunity regulator TBK-1 may contribute to autophagy-mediated elimination of mycobacteria [27]. We hypothesized that inhibition of the STING-TBK-1 mediated immune reaction could explain the AIM2-downregulated autophagy. To test this hypothesis, we examined cell lysates for STING protein expression following infection of BMDMs with *M. bovis* (Figure 5a). The protein level of STING did not appear to alter. We tested the co-localization of STING and *M. bovis* in BMDMs at 24 h post-infection (Figure 5b). In cells transfected with siAIM2, *M. bovis* infection resulted in STING presenting in a punctuated appearance co-localized with *M. bovis*. We also observed more co-localization of TBK-1 and *M. bovis* in cells transfected with siAIM2 (Figure 5c). To examine whether the AIM2 had direct effects on TBK-1, the phosphorylated TBK-1 (p-TBK-1) was detected by western blotting (Figure 5d). We found p-TBK-1 increased markedly and the whole TBK-1 decreased with siAIM2-targeting of transfected BMDMs after infection with *M. bovis*. To investigate this further, we examined whether the absence of AIM2 affected the co-localization of STING and pTBK-1 using immunofluorescence (Figure 5e). The results showed that AIM2 affected the recruitment of TBK-1 by STING. Thus, these data indicated that knock-down of AIM2 promotes the STING-associated signal response during *M. bovis* infection.

### AIM2 inhibits the STING-dependent pathway

**Figure 6:**
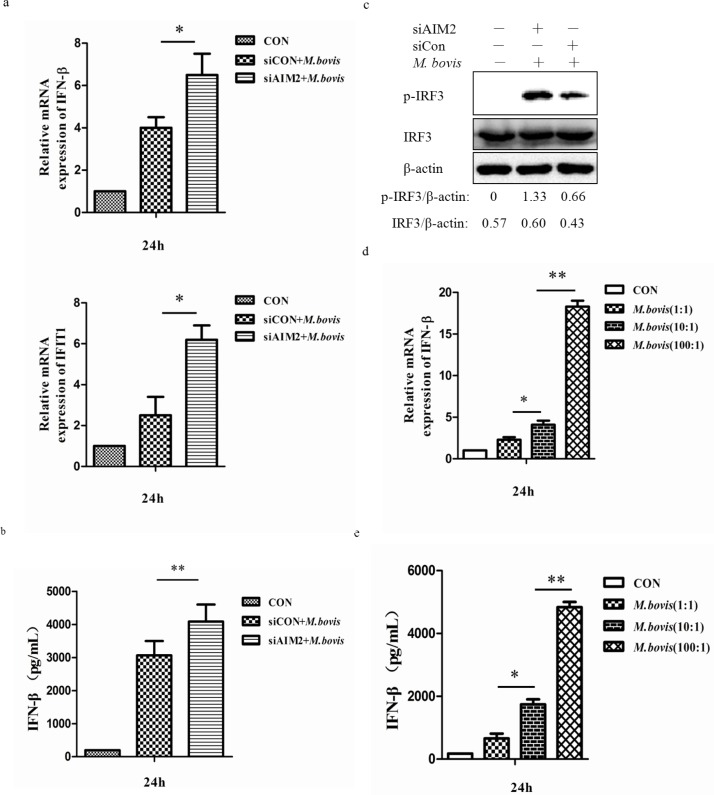
AIM2 inhibits the STING-dependent pathway **a.** mRNA level of IFN-β and IFIT1 were evaluated using quantitative PCR (qPCR) in murine BMDMs transfected with siCon or siAIM2 and then infected 24h with *M. bovis* (MOI 10). **b.** Protein expression of IFN-β was measured by ELISA in murine BMDMs transfected with siCon or siAIM2 and then infected 24h with *M. bovis* (MOI 10). **c.** Protein level of IRF3 and phosphorylated IRF3 (p-IRF3) were analyzed using western blotting in murine BMDMs transfected with siCon or siAIM2 and then infected 24h with *M. bovis* (MOI 10). The IRF3/β-actin and p-IRF3/β-actin ratios are shown below. **d.**, **e.** The expression of IFN-β was measured by qPCR (d) and ELISA (e) in murine BMDMs infected with *M. bovis* at different MOI (1, 10,100) for 24h. *P < 0.05 and **P < 0.01, siAIM2 group *versus* siCON group.

Recent studies have demonstrated that *Mtb* can activate IFN-β transcription through STING-TBK-1-IRF3 pathway [23]. To examine whether AIM2 can affect expression of signal components downstream of STING, we used qPCR to monitor the mRNA expression of IFN-β and IFIT1 and ELISA to test the protein expression of IFN-β in BMDMs 24h post-infection (Figure 6a and 6b). We observed more IFN-β and IFIT1 expression in cells in which the AIM2 was knocked down. Then we tested the phosphorylated IRF3 (p-IRF3) to explain whether the increase of IFN-β was due to the STING-TBK-1-IRF3 axis. There was more phosphorylated IRF3 after AIM2 knock-down in BMDMs (Figure 6c). The siRNA transfection only also has no effect on phosphorylated IRF3 (data not shown). These results confirmed that AIM2 is able to inhibit the STING-TBK-1 mediated immune reaction in BMDMs with *M. bovis* infection.

Then we investigated the effect of bacterial load on the mRNA and protein expression of IFN-βby exposing BMDMs to *M. bovis* at three different MOI (1, 10 and 100) (Figure 6d and 6e), and the results showed a positive correlation between IFN-β and bacterial numbers. This result showed that the expression of IFN-β had the same trend as the change of LC3-II following the amounts of *M. bovis* in the cytosol.

### Exogenous IFN-β induces autophagy

**Figure 7:**
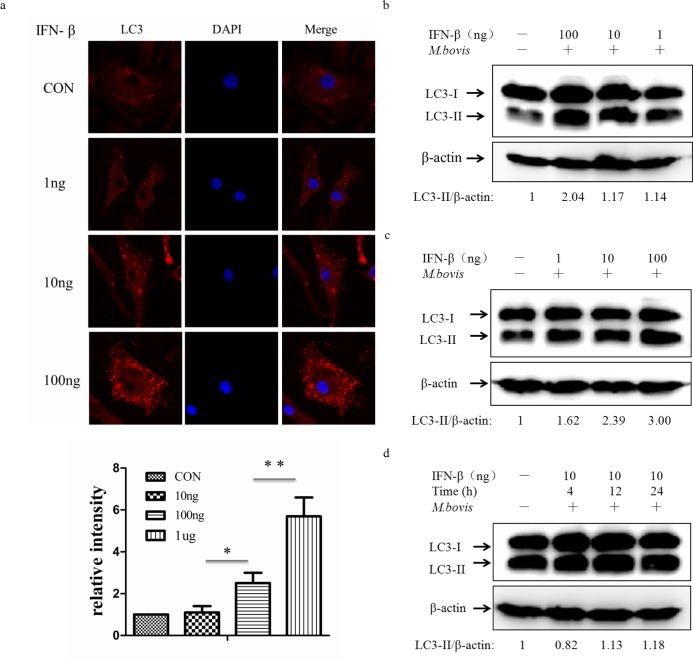
Exogenous IFN-β induces autophagy **a.** Immunofluorescence staining of LC3 (red) in murine BMDMs treated with exogenous murine IFN-β at different doses (1, 10, 100ng) for 24h (above). Nuclei were counterstained with DAPI (blue). The relative fluorescence intensity was shown (*n* = 120) (below). *P < 0.05 and **P < 0.01, IFN-β group *versus* control group. **b.**, **c.** Protein level of LC3 was analyzed using western blotting in murine J774A.1 macrophages (b) or RAW264.7 macrophages (c) treated with IFN-β at different doses (1, 10, 100ng) for 24h. The LC3-II/β-actin ratios are shown below. **d.** Western blot of LC3 in murine J774A.1 macrophages treated with IFN-β at 10ng for different times (4, 12, 24h). The LC3-II/β-actin ratios are shown below. Data were performed three times and expressed as the mean ± SD, and are representative of three separate experiments.

Type I IFNs (IFN-α and IFN-β) are potent antiviral signal molecules, although they inhibit antibacterial signaling pathways by suppressing inflammatory components of Th1-type immunity, such as IL-1α, IL-1β and type II interferon, and promote infection by *Mtb* [23, 28–30]. Less is known about the role of Type I IFNs on autophagy in macrophages, since some researchers have shown that type I IFN-induced autophagy in multiple cancer cell lines and the role was dose-and time-dependent [31]. We therefore treated BMDMs with murine IFN-β at three different doses, and LC3 expression was detected by immunofluorescence at 24 h post-infection (Figure 7a). Treatment of macrophages with IFN-β resulted in more and larger aggregates of LC3. To further validate the role of IFN-β, we treated the immortalized J774A.1 and RAW264.7 macrophage cell lines with exogenous murine IFN-β (Figure 7b and 7c). We also observed the increase of LC3-II in a dose-dependent manner which did not depend on time (Figure 7d). These results showed that IFN-β can active autophagy in a dose-dependent manner.

### AIM2-inhibited autophagy increases *M. bovis* survival *in vitro*

**Figure 8:**
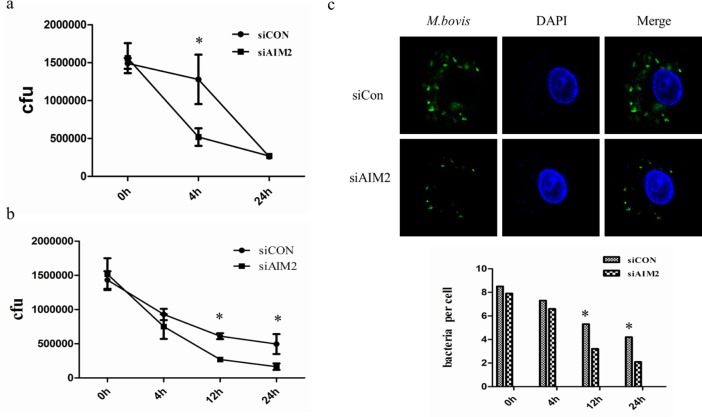
AIM2-inhibited autophagy increases *M. bovis* survival *in vitro* **a.**, **b.** Colony-Forming Units (CFU) from J774A.1 macrophages (a) and BMDMs (b) both transfected with siCon or siAIM2 and then infected with *M. bovis* (MOI = 10). **c.** The number of bacteria was counted in murine BMDMs treated with AIM2 siRNA and then infected with Alexa 488-labeled *M. bovis* (green) (MOI 10) for 0h, 4h, 12h, and 24h. The number of bacteria in macrophages (*n* = 100) was counted and shown below.

The AIM2 inflammasome activation in *M. bovis*-infected macrophages may be induced by cytosolic bacterial DNA released from the phagosome to the cytosol [16]. *Mtb* can also activate the DNA-dependent cytosolic surveillance pathway (CSP) by elicitation of type I IFNs that inhibit the antibacterial signaling pathway and induces autophagy-targeted bacteria for degradation by the host DNA-sensing pathway [13, 23]. Under some circumstances the three innate immunity pathways that are associated with cytosolic DNA during *Mtb* infection may achieve a balance. We have demonstrated that the AIM2 knock-down can increase autophagy and IFN-β secretion by upsetting this balance. We tested bacterial survival in J774A.1 macrophages and BMDMs (Figure 8a and 8b). The results showed that AIM2 knock-down decreased the survival of *M. bovis* in some time in macrophages. Then, we confirmed the survival of *M. bovis* in BMDMs by immunofluorescence (Figure 8c). These data suggest that AIM2 may affect bacterial elimination by inhibiting cytosolic DNA-dependent autophagy during *M. bovis* infection in macrophages *in vitro*.

## DISCUSSION

It has been demonstrated that *Mtb and M. leprae* translocate from the phagolysosome to the cytosol in myeloid cells [20]. Recently, *Mtb*-mediated phagosomal rupture has also been confirmed in mouse spleen and lungs and in numerous phagocyte types [32]. These results imply the significant involvement of the cytosolic innate signaling pathway during mycobacterial infection. There is no doubt that the ESX-1 secretion system which located in RD1 of mycobacteria plays an important role in this process which clearly requires further investigation. To elucidate this process, three new independent studies have explored the involvement of cyclic guanosine monophosphate-AMP (cGAMP) synthase (cGAS) in sensing mycobacterial DNA and its immunological and cellular consequences [33–35]. Although the existence of three pathways, namely the inflammasome [16, 17], autophagy [13] and CSP (cytosolic surveillance pathway) [28] are involved in cytosolic detection, less is known about their relationship. Here we explored the effect of AIM2 on STING-dependent autophagy activation and IFN-β secretion.

Autophagy in natural mycobacterial infection is distinct in different cell types. *Mtb* impairs autophagy at the step of autophagosome-lysosome fusion in human primary dendritic cells (DC) [36] and is also found in human primary macrophages late in infection [37]. These results also indicate the difference between murine and human cells. However, even with cells obtained from the same species studies on autophagy using natural mycobacterial infection can yield different results [13, 38]. Here, our results implied autophagy during *M. bovis* infection is dependent on dose and time and may be induced by the cytosolic DNA from intracellular *M. bovis* based on increasing bacterial numbers in cytosol observed by TEM.

We analyzed the cytosolic DNA sensors that participate in autophagy regulation during microbial infection. As we have shown, the bacteria that escaped from the phagosome to the cytosol may be the source of cytosolic DNA that triggers the AIM2 inflammasome activation in *M. bovis*-infected macrophages [16, 39]. Given the two pathways that are associated with cytosolic dsDNA, we hypothesized that the AIM2 inflammasome activation takes part in inducing autophagy during infection with *M.bovis*. To verify this hypothesis, we carried out the present work and found the activation of autophagy during infection of *M. bovis* did not require the AIM2 inflammasome, on the contrary, it is interesting to note that the siRNA-mediated disruption of AIM2 significantly increased autophagy and also found an increase of the co-localization of selective autophagy marker p62 and LC3 with *M.bovis*. Considering that the inflammasome is a multi-protein complex and the AIM2 knock-down may affect the normal function of its downstream signal molecules, we verified that the inhibition of autophagy in response to *M. bovis* infection was dependent on the inflammasome sensor but did not require complex assembly of the inflammasome, by using caspase-1 inhibitor Z-YVAD-fmk, ASC targeting siRNA and IL-18 and IL-1β neutralizing antibody. Other aspects of the interaction between AIM2 and *M. bovis*, besides the inflammasome sensor function, certainly require further investigation.

Then, we detected another molecular, MITA/MPYS/ERIS/TMEM173 (STING), which has been demonstrated to be involved in autophagy during *Mtb* infection [13]. We hypothesised that inhibition of autophagy by AIM2 may be connected with STING, since both of them can recognize dsDNA in the cytoplasm. Indeed, we showed that siRNA-mediated silencing of AIM2 significantly increased the co-localization of *M. bovis* and STING. Similarly, we also observed an increase in co-localization of *M. bovis* and TBK-1 as TBK-1 has been demonstrated to promote autophagy-mediated antimicrobial defense [27]. This suggests that AIM2 may have a negative role on STING-dependent autophagy in *M. bovis*-infected macrophages. Besides autophagy, activation of cytosolic receptors induces signaling through the STING/TBK-1/IRF3 axis, also resulting in IFN-β production [23, 40, 41]. To confirm the results, the AIM2 knock-down showed a high mRNA level of IFN-β and interferon-induced protein with tetratricopeptide repeats 1, IFIT1. Taken together, we believe that the AIM2 does affect the STING-dependent pathway during *M. bovis* infection.

Type I interferons (IFN) are pleiotropic proteins with anti-proliferative, antiviral, and immunomodulatory activities. Reports showed conflicting results whether the IFN-α/β response is detrimental or beneficial to the host during tuberculosis [42, 43]. For example, IFN-α/β inhibits Th1 type immunity in mice infected with virulence *Mtb* clinical isolate and impairs the ability of human macrophages to control growth of *M. bovis* BCG [44, 45]. In contrast, mice which IFN-α receptor knockout enhances resistance to tuberculosis suggesting that Type I IFN responses are counterproductive in tuberculosis [46]. Recent studies in several cancer cell types showed type I IFN-induced autophagy using autophagic markers [31]. Here, we showed that IFN-β also actives autophagy in a dose-dependent manner as in some cancer cells, but was not time-dependent. This result improved our understanding of type-I IFN in immunity, but has also left unanswered questions. For example, whether the autophagy induced by long term exposure to IFN-β can also inhibit mycobacterial survival and how IFN-β induces autophagy in macrophages. Further work is required to clarify these points.

Finally, we reported a decrease in the survival of *M. bovis* in macrophages by using siAIM2. Our results were different to previous studies where AIM2 has been shown it's favorable for the host by inhibiting *Mtb* survival *in vivo* [17]. This may attribute to the difference between the two mycobacterial species, or the difference regulatory system *in vitro* and *in vivo*. We consider the inhibition of *M. bovis* may be due to the increased autophagy after AIM2 knock-down. Taken together, our results demonstrated that the AIM2 induced by a virulent strain of *M. bovis* in murine macrophages led to an inhibition of autophagy activation, and this inhibition is due, at least in part, to restriction of *M. bovis*-induced STING-TBK1-dependent autophagy activation and IFN-β production by conjugating competitively with cytosolic *M. bovis* DNA.

## MATERIALS AND METHODS

### Reagents

The rabbit anti-LC3B antibody (L7543) was from Sigma-Aldrich. The rabbit SQSTM1 (p62) antibody (AP2183b) was from Abgent (San Diego, CA, USA). The rabbit polyclonal anti-mouse AIM2 antibody (sc-137967) and rabbit polyclonal anti-mouse ASC antibody (sc-22514-R) and the goat polyclonal antibody TMEM173 (M-12) (sc-241049) were from Santa Cruz Biotechnology (Santa Cruz, CA, USA). The rabbit anti-mouse β-actin (AP0060) was obtained from Bioworld Technology (Nanjing, Jiangsu, China). The rabbit Anti-NAK/TBK-1 antibody (ab40676) and rabbit anti-NAK/TBK-1 (phosphor S172) antibody (ab109272) were obtained from Abcam (Cambridge, UK). The rabbit TMEM173 polyclonal antibody (19851-1-AP), rabbit IRF3 polyclonal antibody (11312-1-AP) and rabbit IL-1β polyclonal antibody (16806-1-AP) were from Protein Tech (Wuhan, Hubei, China). The phosphor-IRF3 (Ser396) (4D4G) was from Cell Signaling Technology (Boston, Mass, USA). The goat anti-rabbit secondary antibody and donkey anti-goat secondary antibody were obtained from Santa Cruz Biotechnology, Beijing ZSGB Biotechnology and Beijing Cowin Biotechnology (Beijing, China), respectively. The Fast Protein Precipitation and Concentration Kit were purchased from Beyotime Institute of Biotechnology (Shanghai, China). Reagents and apparatus used in immunoblotting assays were obtained from Bio-Rad (Hercules, CA, USA). The recombinant mouse IFN-β was from ProSpec (Rehovot, Israel) and mouse M-CSF were from Pepro Tech (Rocky Hill, NJ, USA). Caspase-1 specific inhibitor, z-YVAD-fmk, was from BioVision (Palo Alto, CA, USA). The mouse interferon-β ELISA kit (CSB-E04945m) was from Cusabio (Wuhan, Hubei, China)

### Cell culture

Mouse macrophage cell lines J774A.1 and RAW264.7 were obtained from Cell Culture Center, Xiehe Medical University (Beijing China), and were cultured as described before [16]. Mouse bone marrow cells (BMDMs) were isolated from femurs of female 6-8 week old C57BL/6 mice as described previously [16], and cultured in circular cell culture dish (Corning, New York, USA) for 7d in RPMI1640 (Hyclone, Logan, UT, USA) supplemented with 10 ng/ml M-CSF (Pepro Tech), 10 % fetal bovine serum (FBS) (Gibco, Grand Island, NY, USA), 100 ug/ml streptomycin and 100 U/ml penicillin (Gibco).

### Bacterial culture and infections

Virulent *M. bovis* Beijing strain was obtained from China Institute of Veterinary Drug Control (CVCC, China). Bacteria were cultured from frozen stocks in 7H9 Middlebrook media (BD Biosciences) containing albumin-dextrose-catalase (ADC) enrichment solution and 0.05 % Tween-80 (Difco) and grown to mid-logarithmic phase for 1 week at 37°C.

J774A.1 and RAW264.7 macrophages and BMDMs were infected with *M. bovis* at 37°C with 5 % CO_2_. Cells were washed three times with warm PBS to remove extracellular bacteria after two hours. All experiments were performed for three times.

### Small interfering RNA (siRNA) transfections and treatments

Mouse AIM2-targeting siRNA oligonucleotides (ON-TARGET plus SMART pool) were purchased from Dharmacon (Waltham, MA, USA). The sequences of each siRNA oligonucleotide in this pool are as follows: mAim2 J044968-09, 5′-ACAUAGACACUGAGGGUAU-3′; mAim2 J044968-10, 5′-UGUCUAAGG CUUGGGAUAU -3′; mAim2 J044968-11, 5′-CUACCUGAGGAUAGCAUUU-3′ and mAim2 J044968-12, 5′-AGUACUAAGA A AUCAGUGA-3′. Non-targeting control siRNA oligonucleotides (ON-TARGET plus NON-targeting Pool) were also obtained from Dharmacon. Mouse ASC-targeting siRNA oligonucleotide was obtained from Qiagen (GS66824). For siRNA transfection, cells were seeded in 24-well plates at a density of 1×10^5^ cells/well, and then transfected with siRNA oligonucleotides (50 nM) using 3 μL HiperFect Transfection Reagent (Qiagen, Valencia, CA, USA). The decrease in AIM2 and ASC expression was checked by qRT-PCR and western blot analysis.

### RNA isolation, complementary DNA synthesis and quantitative real-time polymerase chain reaction (PCR)

Total RNA was performed by SV Total RNA Isolation System (Promega, Madison, WI, USA), and reverse transcription was performed by the Revert Aid first-strand cDNA synthesis Kit (Fermentas, Glen Burnie, MD, USA) according to the manufacturer's instructions. Quantitative PCR was performed using the DNA Engine Opticon TM2 fluorescence detection system (MJ Research Inc., Waltham, MA, USA) and SYBR Green Master Mix (Bio-Rad). The special gene primer pairs are shown in Table 1. Quantitative PCR data were analyzed using the comparative CT method (2-ΔΔCT). All samples were analyzed in triplicate [21].

**Table 1:** Primers used for quantitative real-time PCR

Gene	Forward primer (5′-3′)	Reverse primer (5′-3′)
**IFN-β**	AAGAGTTACACTGCCTTTGCCATC	CACTGTCTGCTGGTGGAGTTCATC
**IFIT1**	GCCTATCGCCAAGATTTAGATGA	TTCTGGATTTAACCGGACAGC
**β-actin**	CCTTCTGACCCATTCCCACC	GCTTCTTTGCAGCTCCTTCG

### Isolation of bacterial DNA from cytosolic fraction and polymerase chain reaction (PCR) amplification

BMDMs were infected with *M. bovis* at MOIs of 10. After 2 hours, cells were washed vigorously with warm PBS three times, and the cytosolic fraction was then isolated by the Qproteome Cell Compartment Kit, following the manufacturer's instructions (Qiagen).

To extract bacterial DNA, *M. bovis* (5 × 10^7^colony-forming units (CFU)) was bathed water at 80°C for 30 minutes and supernatants were collected by centrifugation.

The PncA gene was amplified by PCR using the specific primers that designed according to the published sequence of PncA gene of *M. bovis*. A 294bp fragment was specifically amplified from *M. bovis*. PCR amplification was performed with GoTaq DNA Polymerase (Promega) and 20ng of each DNA as template. (Forward primer: CTCAGCTGGTCATGTTCCCCAT, Reverse primer: CGGTGTGCCGGAGAAGCCG)

### Immunobloting analysis

Total cell proteins were extracted using a protein extract kit (Fast Protein Precipitation and Concentration Kit, Wuhan Boster Biotech, Wuhan, China), and then were mixed with 5xSDS sample buffer. The mixtures were separated by different SDS-PAGE (8 %-15 %according to the molecular weight) and analyzed by immunoblot as described previously [16]. The image J software was used to analyze the intensity.

### ELISA

ELISA for IFN-β was performed with the macrophage cell culture supernatants by using mouse interferon-β ELISA kit as per manufacturers’ instructions.

### Immunofluorescence assay

10 mL of a log-phase *M. bovis* culture were transferred into a 15-mL conical tube. Mycobacterial cultures were centrifuged at 3000 rpm for 5min, the supernatant fraction was carefully removed and the pellet washed twice with 10 mL of 1x PBS. This was centrifuged at 3000 rpm for 5min and the supernatant fraction removed and discarded. The pellet was resuspended in 1 mL of 1x PBS and the cell suspension transferred into a 1.5-mLmicrocentrifuge tube. 10 ul of Alexa 488 caboxylic acid succinimidyl ester stock solution was added to the tube to make a final concentration of 10 mg/ml. The tube was wrapped with foil and incubated at 37°C for 45-60 min on a shaker. Mycobacteria were pelleted by centrifugation at 10000 rpm for 3 min at room temperature (RT). The supernatant was removed and washed twice with 1 mL of 1x PBS. The mycobacterial pellet was resuspended in 6 mL of complete DMEM.

All reagents for fixation, wash and blocking were purchased from Beyotime (Beyotime Institute of Biotechnology, China). Cells were grown on poly-lysine-coated coverslips. After treatment, cells were fixed in an Immunol-staining fix solution (Beyotime) for 10 min at RT and washed three times for 5 min with PBS. Then they were blocked for 1 hour at RT. Rabbit anti-LC3B (1:200), rabbit anti-SQSTM1 (1:200), rabbit anti-NAK (1:200), rabbit anti-IRF3 (1:200), and goat anti-TMEM173 (1:50) were added, respectively, and incubated at 4°C overnight and washed three times with PBS for 5 min. Goat anti-rabbit IgG Alexa Fluor 594 (1:200), FITC-conjugated antibody (1:200), and donkey anti-goat IgG Alexa Flour 647 (1:200) were added and incubated at 37°C for 1 h and washed three times with PBS for 5 min. DAPI (1:10) was added and incubated at RT for 5 min and washed three times with PBS for 5 min. The macrophages were finally mounted with glycerin buffer and examined immediately with an OLYMPUS microscope.

### Transmission electron microscopy

Infection of J774A.1 macrophages with *M. bovis* (MOI 1, 10 and 100) was carried out as described above. After24h, cells were scraped and centrifuged for 5 min at 1000 g. Cell pellets were fixed in 2.5 % glutaraldehyde in 0.2 M phosphate buffer (pH 7.4). Fixed cells were post-fixed in 2% osmium tetroxide and 100 mM cacodylate buffer, dehydrated with increasing concentrations of ethanol and gradually infiltrated with Epon resin (Pelco). Thin sections were stained with uranyl acetate and lead citrate and examined using transmission electron microscope.

### Colony-forming unit assay

J774A.1 cells were plated in 12-well plates (5×10^5^ cells/well). Cells were infected with 5×10^6^cells per mycobacteria for 2hr at 37°C,washed three times with PBS. The CFU was determined by plating 100 μL of serial dilutions onto plates containing Middlebrook 7H11 agar, supplemented with ADC enrichment solution and 0.05 % Tween-80 (Difco).

The rest of information about reagents, bacterial culture and infections, small interfering RNA (siRNA) transfections, RNA isolation, complementary DNA synthesis and quantitative real-time polymerase chain reaction (PCR), isolation of bacterial DNA and PCR amplification, immunobloting analysis, immunofluorescence assay, transmission electron microscopy, and colony-forming unit assay are presented in Supplementary methods.
